# The Roles of Myeloid Cells in Aging-related Liver Diseases

**DOI:** 10.7150/ijbs.82352

**Published:** 2023-03-05

**Authors:** Jinghao Yao, Yang Li, Hua Wang

**Affiliations:** 1 Department of Oncology, the First Affiliated Hospital of Anhui Medical University, Hefei 230022, China; 2 Inflammation and Immune Mediated Diseases Laboratory of Anhui Province, Anhui Medical University, Hefei 230032, China; 3 Department of Genetics, School of Life Science, Anhui Medical University, Hefei 230032, China

**Keywords:** Aging, Myeloid Cells, Microenvironment, Inflammation, Liver Disease

## Abstract

Aging is a necessary process of life associated with various mechanisms, such as genomic instability, loss of proteostasis, deregulated nutrient sensing, and cellular senescence, causing progressive dysregulation of the microenvironment, organ homeostasis and biological functions. The hepatic microenvironment is essential for maintaining liver homeostasis, in which hepatocytes, sinusoidal endothelial cells, stellate cells and immune cells are closely associated with the development of aging-related liver diseases. There is increasing evidence that immunocytes, especially myeloid cells, are involved in aging-related liver diseases such as alcoholic liver disease, nonalcoholic liver disease, liver fibrosis or cirrhosis and liver cancer, becoming promising treatment targets of these diseases. This review summarizes the phenotypic and functional alterations associated with aging liver and myeloid cells, as well as the roles of myeloid cells in the progression of aging-related liver diseases.

## Introduction

Aging is commonly characterized as an organism's functional decline over time, with characteristics such as loss of physiological integrity, disruption of organ homeostasis, and progressive degradation of many biological systems 1. These changes reshape the immune landscape in both physiological and pathological states, increasing the susceptibility of the organism to pathogenic factors 2. With the improvement in living standards and advances in medicine, human life expectancy is significantly increased, leading to the prominent aging of the population accompanied by various chronic diseases, which demonstrate a natural relationship between aging and the pathogenic processes of many chronic diseases 3, 4.

Liver diseases cause approximately 1.2 million deaths each year, accounting for 3.5% of deaths and affecting the lives of 1.5 billion people worldwide 5. The liver is a complex metabolic organ that maintains the homeostasis of the body by regulating energy metabolism, biosynthesis, and xenobiotic removal 6-8. The liver is also an important immune organ, where a variety of immune cells are temporarily or permanently distributed to perform immune surveillance of pathogens of intestinal origin as well as senescent cells 7. The aged liver typically exhibits a higher infiltration of immune cells such as macrophages, neutrophils, lymphocytes and natural killer (NK) cells, resulting in a higher inflammatory state 9 which aggravates liver aging and increases the susceptibility to aging-related liver diseases, including nonalcoholic fatty liver disease (NAFLD), alcoholic liver disease (ALD), liver fibrosis or cirrhosis, and liver cancer 10. Among the immune cells, myeloid cells such as macrophages and neutrophils play major roles in the development of liver diseases independently or through crosstalk with nonimmune mesenchymal cells 11. However, the specific mechanisms by which myeloid cells induce aging-related liver diseases remain largely unclear.

The incidence of aging-related liver diseases will continue to rise as the population ages, which poses new requirements for further in-depth studies on the mechanisms between myeloid cells and liver aging. This review summarizes the phenotypic and functional alterations of aging liver as well as myeloid cells and presents the latest research progress of myeloid cells in affecting aging-related liver diseases, which will provide new insights for further exploration of the clinical strategies of aging-related liver diseases.

## Aging liver

**Figure 1:**
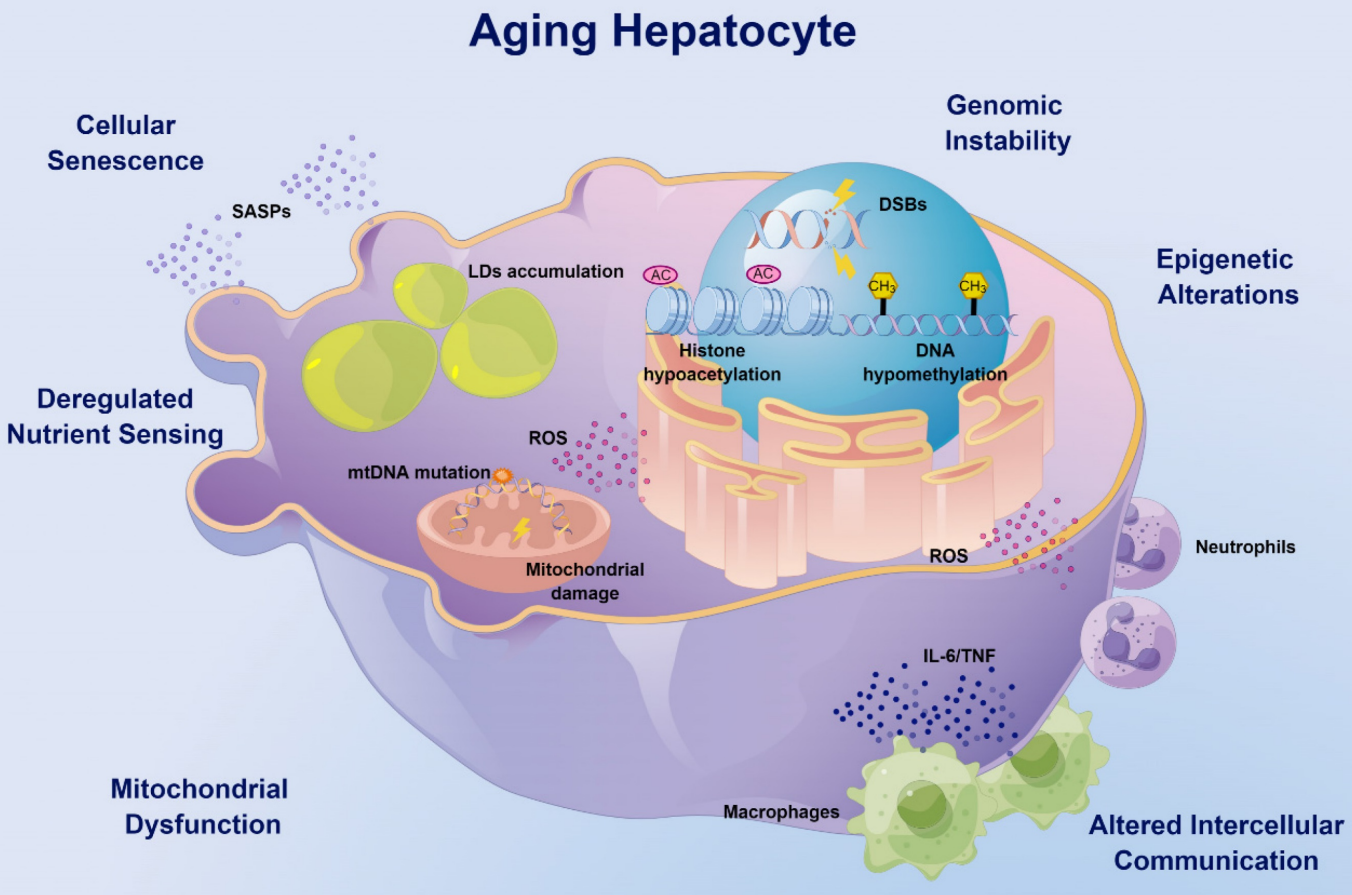
The hallmarks of aging hepatocytes. Aging hepatocytes encompass most of the hallmarks of aging. With age, DNA damage such as DSBs can occur spontaneously or be induced by ionizing radiation or ROS, causing pathological features of aging. Epigenetic alterations such as DNA methylation and histone acetylation were significantly reduced in aged livers, which possibly contributed to aging-related metabolic dysfunction. Deregulation of the trophic and metabolic pathways is another hallmark of aging hepatocytes, and decreased expression of MANF may promote lipid accumulation in the aged liver. Mitochondria in hepatocytes are also altered with age and are associated with a decrease in mitochondrial membrane potential and an increase in volume and peroxide production, supporting the key role of mitochondrial damage during aging. Senescent hepatocytes significantly accumulate in aged liver due to the accumulation of DNA damage and activation of the p53-p21 and p16^ink4a^-pRb pathways. In addition, abnormal intercellular communication between hepatocytes and immune cells such as macrophages and neutrophils can cause persistent sterile inflammation, known as inflammaging. DSBs: DNA double-strand breaks; MANF: mesencephalic-astrocyte-derived neurotrophic factor; ROS: reactive oxygen species.

Aging liver encompasses most of the hallmarks of aging (Figure 1) and is associated with progressive changes in hepatic structure and function, as well as various hepatocyte alterations, including reduced proliferative capacity, metabolic and nutritional imbalances and altered liver microenvironment, which ultimately leads to increased disease susceptibility.

The liver is the most resilient organ in adults and is characterized by its unique regenerative capacity. From their maturity, the volume of hepatocytes starts to decrease gradually with age 4. On the one hand, senescent hepatocytes accumulate in the liver as a result of DNA damage accumulation and the activation of the p53-p21 and p16^ink4a^-pRb pathways 12, 13. On the other hand, DNA double-strand breaks (DSBs) 14 and mutations in mitochondrial DNA (mtDNA) 15 caused by ionizing radiation or reactive oxygen species (ROS) may cause aging phenotypes of the liver, including expression of aging markers, mitochondrial fusion and elongation, and altered gene expression profiles. Moreover, the process of liver diseases and natural aging is accompanied by increased hepatocyte polyploidy 13, 16, which limits the ability of hepatocytes to proliferate and may affect the recovery from inflammatory liver injury and partial hepatectomy 17.

Deregulation of the trophic and metabolic pathways is another hallmark of the aging liver, which significantly affects liver morphology, physiology, and oxidative capacity. Adenosine 5'-monophosphate activated protein kinase (AMPK), a serine-threonine kinase, is essential for regulating the homeostasis of energy metabolism in cells, and phosphorylated AMPK (activation) stimulates the physiological process of energy production and inhibits energy utilization 18. The AMPK signaling declines with age in a variety of tissues, thus affecting mitochondrial function and energy homeostasis 19. Interestingly, although increased levels of phosphorylated AMPK can be observed in the aging liver, the signaling pathway is impaired because of the inactivated downstream signals 20. Multiple approaches including exercise, dietary interventions and probiotic supplementation can improve liver aging and steatosis by activating the AMPK pathway 21, 22. Moreover, decreased expression of mesencephalic astrocyte-derived neurotrophic factor (MANF) with age induces alterations in the transcriptome of lipid metabolism genes in mouse hepatocytes, promotes lipid accumulation in the liver through the induction of G0/G1 switch gene 2 (G0S2), and causes hepatic steatosis in response to metabolic stresses such as a high-fat diet (HFD) 23. As the metabolic center, aging-related mitochondrial damage may decrease the metabolic capacity of hepatocytes. Recent studies have shown that the Hedgehog pathway is downregulated with age, leading to a decrease in the quantity and function of mitochondria, which results in reduced hepatocyte elasticity, impaired liver regeneration, and greater susceptibility to inflammation 24. Epigenetic alterations, including DNA methylation and histone acetylation, may also affect liver metabolism 25-27. Histone hypoacetylation is upregulated with age, leading to impaired hepatocyte plasticity, and selective inhibition of histone deacetylase (HDAC) can significantly restore plasticity and repopulation of aged hepatocytes confronting liver injury 28, 29. Our previous studies have revealed that sirtuin-1 (SIRT-1), another class of histone deacetylases with antiaging effects 30, 31, is downregulated with age and increases the susceptibility of hepatocytes to alcohol damage 32. The different regulation between SIRT-1 and HDAC may reflect the functional diversity of acetylation modification.

The hepatic microenvironment comprises mainly liver sinusoidal endothelial cells (LSECs) 33, hepatic stellate cells (HSCs) 34 and Kupffer cells (KCs) 35, which synergistically influence the aging process of the liver. Pseudocapillarization, a typical morphological change in aging LSECs 36, results in drug metabolism impairment 33 and lipoproteins 37 and insulin clearance 38, causing hyperlipidemia and hepatic insulin resistance, which may lead to cardiovascular and metabolic diseases. Hepatocyte growth factor (HGF) released by HSCs is significantly reduced with age, resulting in decreased regenerative capacity of the liver 34. KCs are liver resident macrophages 39, the number of which gradually increases with age 40. Autophagy and autophagy-associated protein 5 (ATG5) expression is reduced in KCs with age, promoting their polarization toward the pro-inflammatory M1 phenotype 35. In aged rats, the numbers of both pro-inflammatory M1 and anti-inflammatory M2 KCs have been reported to be increased 41, which may reflect the continued activation of the immune response in advanced age.

For healthy aging, alterations in hepatocytes themselves reduce the proliferation and function, while the roles of pathological factors such as inflammation in liver aging have been understudied. As an important immune organ, in addition to KCs, a variety of immune cells also colonize the liver. Aging not only leads to a decrease in the defensive capacity of the liver but is also accompanied by greater inflammatory injury, known as inflammaging. Myeloid cells, including macrophages and neutrophils, have been reported to play significant roles in inflammatory liver injuries 42, which may be exacerbated by aging. Therefore, this review will further focus on the impact of aging immune cells, especially myeloid cells, on liver aging and disease susceptibility.

## Aging myeloid cells

Aging of the immune system, which refers to an age-related decline in immune surveillance and clearance, dramatically increases the susceptibility to diseases 43. Inflammaging, one of the hallmarks of immune aging, is the chronic progressive elevation of a low inflammatory response in naturally aging organisms in the absence of pathogens 44. A major feature of inflammaging is the chronic activation of the innate immune system 45 with an imbalance in the secretion of pro- and anti-inflammatory factors 46, which may drive the aging of solid organs 47.

**Figure 2:**
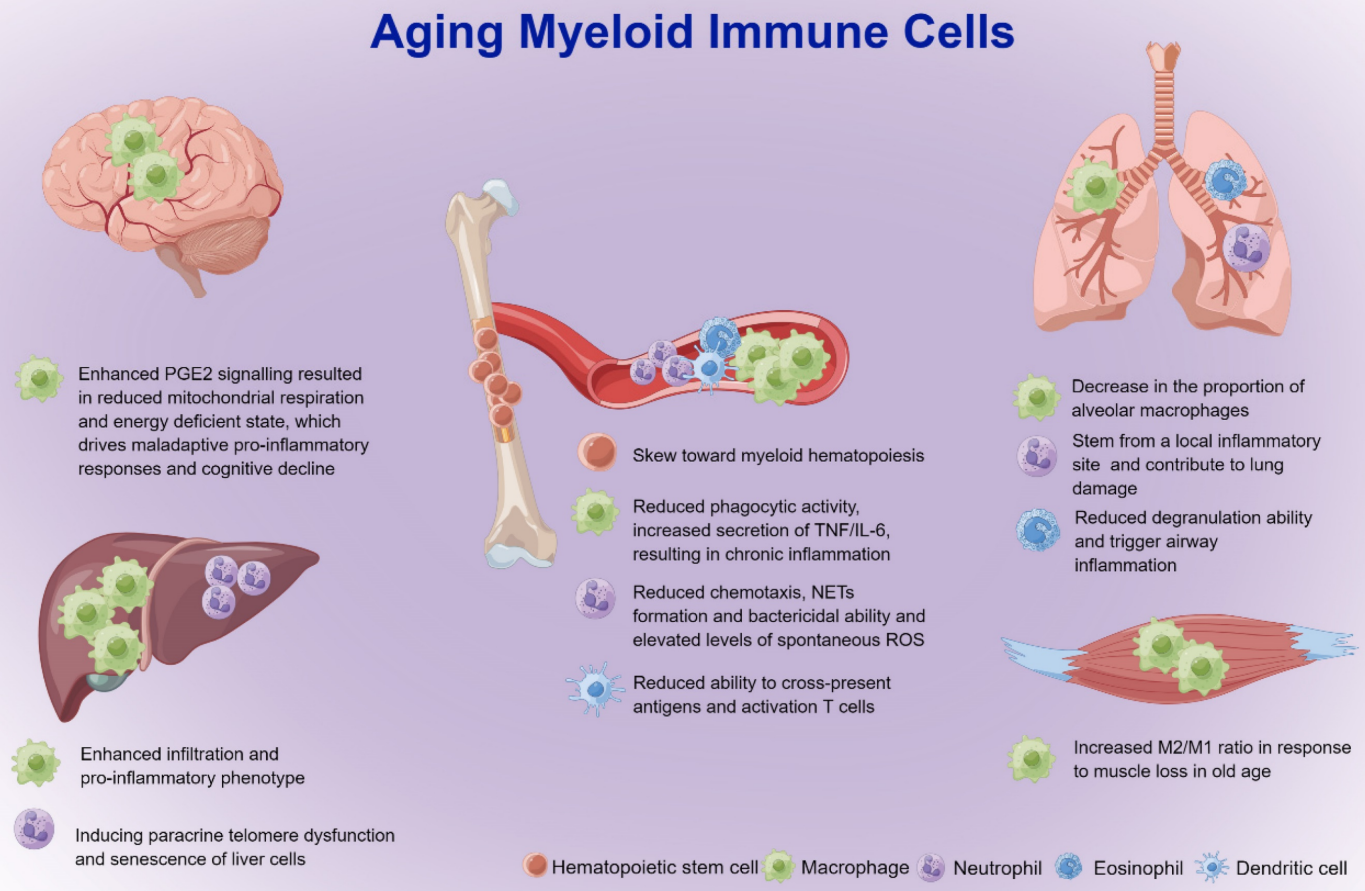
Effect of myeloid cells on aging-related organ injuries and diseases. Hematopoietic stem cells in the aged body exhibit reduced output of lymphoid and erythroid lineages but relatively more output of myeloid lineage cells than those in the young body. With age, macrophages with reduced phagocytic activity and increased secretion of TNF/IL-6 result in polarization to the pro-inflammatory M1 phenotype as well as chronic inflammation in the liver and central nervous system, and the anti-inflammatory M2 phenotype in muscles. Aging neutrophils may induce paracrine telomere dysfunction and senescence of liver cells, and loss of neutrophil-directed motility at epithelial junctions may lead to re-entry of neutrophils into the circulation and cause distant lung tissue injury. The degranulation capacity of eosinophils is significantly reduced in elderly individuals, which may trigger airway inflammation. Aging DCs are significantly less efficient in cross-presenting antigens and activating CD8^+^ T cells, which may lead to a decrease in adaptive immune responses in aged hosts. DCs: dendritic cells; IL-6: interleukin-6; M1: M1 macrophages; M2: M2 macrophages; NETs: neutrophil extracellular traps; PGE2: prostaglandin E2; ROS: reactive oxygen species; TNF: tumor necrosis factor.

The functional integrity of the immune system is shaped by hematopoietic stem cells, the source of immune cells 48. Aging hematopoietic stem cells exhibit reduced output of lymphoid and erythroid lineages, whereas output of myeloid lineage is maintained or even increased compared with young hematopoietic stem cells 49. This skew toward myeloid hematopoiesis appears to be related to early differentiation of hematopoietic stem cells, as old mice have increased numbers of common myeloid progenitor cells (CMPs) and decreased numbers of common lymphoid progenitor cells (CLP) compared to young mice 50, 51. Myeloid cells include mainly monocytes/macrophages, neutrophils, eosinophils, basophils, myeloid-derived suppressor cells (MDSCs) and dendritic cells (DCs). Aging increases the accumulation of myeloid cells and causes a significant reduction in the capacity of movement and surveillance, which further leads to organ injuries and diseases (Figure 2) 52-54. Therefore, further studies focusing on aging myeloid cells in specific inflammatory settings are needed to better understand how such cells contribute to organ damage and diseases in the elderly population.

### Macrophages

Macrophages are versatile immunocytes that perform critical roles in innate immunity, inflammation and tissue homeostasis 55, 56. Macrophages can be classified as either tissue-resident or monocyte-derived populations. Tissue-resident macrophages, such as KCs, are differentiated from erythro-myeloid progenitors (EMPs) in the yolk sac and fetal liver, while monocyte-derived macrophages are differentiated from bone marrow 57. During inflammation or tissue injury, depending on the tissue type and stimuli, both resident and monocyte-derived macrophages engage in migration, expansion, and signaling. During extravasation into inflammatory sites under chemokine guidance 58, monocytes differentiate into macrophages to perform immune functions, largely based on various signaling molecules presented in the local microenvironment 59. Depending on the stimulus, macrophages can be classified as the pro-inflammatory M1 phenotype, which is polarized by either lipopolysaccharide (LPS) alone or in combination with T helper 1 (Th1) cell cytokines, or the anti-inflammatory M2 phenotype, which is polarized by T helper 2 (Th2) cell cytokines such as interleukin-4 (IL-4) 60, 61. However, the scenario *in vivo* is often more complex, and they are generally accepted as transient, reversible, and occurring along a spectrum 62.

The number of monocytes in the bone marrow and peripheral circulation rises with age, creating chronic inflammation by secreting more cytokines such as tumor necrosis factor (TNF) and IL-6 63. Macrophages are found to infiltrate more in liver and white adipose tissue with age 64 but less in bone and skeletal muscle 65, 66. Aged macrophages have a significantly altered transcriptional profile and often exhibit a stronger pro-inflammatory phenotype (M1) than younger macrophages 64, 65. Therefore, the M1/M2 ratio could reflect the age-related functional status of macrophages.

The phagocytic capacity of macrophages decreases with age, which reduces bacterial killing and inflammatory clearance. 1A/1B-light chain 3 (LC3)-associated phagocytosis (LAP) is an important innate immune response of macrophages 67. Bone marrow-derived macrophages (BMDMs) in aged mice not only lack LAP and bacterial killing but also produce higher levels of pro-inflammatory cytokines, resulting in susceptibility to pathogens 67. Increased macrophage p38 mitogen-activated protein kinase (MAPK) activity in elderly individuals leads to a decrease in T-cell immunoglobulin mucin receptor-4 (TIM-4) on macrophages, causing reduced efferocytosis of apoptotic vesicles 68. Therefore, although the onset of inflammation is similar between young and elderly individuals, the ability to eliminate inflammation is greatly diminished in elderly individuals, resulting in persistent chronic inflammation.

Recent studies have highlighted the critical role of metabolism in the programming of the immune response 69. In aging macrophages and microglia, prostaglandin E2 (PGE2), a typical senescence-associated secretory phenotype (SASP) 70, signals through the EP2 receptor to promote glycogen synthesis and reduce glucose flux and mitochondrial respiration 71. This energy-deficient state triggers an undesirable pro-inflammatory response. The NAD^+^ of macrophages is derived mainly from the kynurenine pathway metabolism of tryptophan. Although the immune response triggers upstream kynurenine pathway activation, aging inhibits NAD^+^ de novo synthesis in macrophages and suppresses mitochondrial NAD^+^-dependent signaling and respiration, which shift macrophages toward a pro-inflammatory phenotype and impair phagocytosis and resolution of inflammation 72.

There is great heterogeneity of macrophages from different organs during aging 73-75. Single-cell RNA sequencing (scRNA-seq) analysis revealed a significant increase in the proportion of interstitial lung macrophages but a decrease in the proportion of alveolar macrophages. There was an increase in the proportion of hepatic macrophages expressing chemokine (C-X3-C motif) receptor-1 (CX3CR-1), but the number of peritoneal macrophages expressing intercellular adhesion molecule 2 (ICAM2) was significantly reduced 74. In addition, scRNA-seq analysis of aged mice showed enhanced inflammatory function of KCs, which may exacerbate inflammatory injury in the liver 75. Recent research revealed that the number and transcriptional characteristics of alveolar macrophages that change with age are not cell autonomous, but rather are formed by the alveolar milieu in which they live, irrespective of signaling molecules or cells in the circulation 76. This finding emphasized that the local microenvironment of tissues may play a dominant role in macrophage aging.

Although aging is irreversible, the inflammatory state of an aging organism can be improved to some extent by reducing food intake and avoiding malnutrition 77. Recent studies revealed that caloric restriction (CR) 64 and fibroblast growth factor-21 78 could regulate macrophage polarization from the M1 to M2 phenotype in several organs of an aged mouse model, thereby suppressing the persistent inflammation associated with aging. Therefore, strategies targeting macrophages may extend human lifespan and improve susceptibility to aging-related diseases.

### Neutrophils

Neutrophils, the most prevalent leukocytes in the body, serve as the first line of defense against pathogens and respond to a variety of inflammatory stimuli 79. Neutrophils originate from CMPs in the bone marrow and extramedullary tissues such as spleen 79. After differentiation into granulocyte macrophage progenitor cells (GMPs) 80, neutrophil-committed differentiation starts with neutrophilic promyelocytes and myelocytes, which both have the propensity to divide and are part of the so-called “mitotic neutrophil pool”. The presence of mature neutrophils in the bone marrow represents the end of the “postmitotic pool”, where neutrophils undergo terminal differentiation before being liberated into the peripheral blood. Then, mature neutrophils in the “mature neutrophil pool” enter a state of exchange between the bone marrow, blood, and other tissues 81. When stimulated by pathogens, neutrophils in blood can rapidly migrate to the site of infection through cytokine-mediated chemotaxis 82, β2 integrin-mediated vascular endothelial adhesion, ICAM-1- and ICAM-2-mediated transendothelial migration 83, and defend against invading pathogens 84 through the release of cytotoxic proteins, peptides and enzymes in the phagolysosome 85, ROS 86 and neutrophil extracellular traps (NETs) formation (NETosis) 87. Eventually, neutrophils that have engulfed bacteria accumulate in the liver and are taken up by KCs, which reduces the generation of inflammatory cytokines by macrophages and promotes inflammation resolution once infections have been removed 88. However, if pathogens are not cleared promptly, neutrophils may induce or aggravate many inflammatory diseases, including both acute and chronic diseases pertaining to the liver, lung and other organs 89, 90.

During the aging process, neutrophil infiltration is increased in a variety of organs 64, but neutrophil chemotaxis 91, phagocytosis and bacterial killing activity are significantly reduced 92. This decrease in chemotactic directionality causes neutrophil elastase-mediated tissue degradation and neutrophil-mediated local inflammation, leading to additional tissue injury 93, 94. Neutrophils produce a burst of ROS that can directly kill pathogens. Compared to neutrophils in younger individuals, acute ROS generation is substantially lower in older individuals 95, whereas spontaneous ROS is significantly higher 96, 97, which may indirectly lead to persistent inflammation and a reduced immune response to pathogens. Aging can also cause excessive neutrophil activation, which is involved in liver injury and mortality associated with viral infection by inducing IL-17 production from natural killer T (NKT) cells 98. In addition, NETosis is the ability of neutrophils to expel genomic DNA to capture pathogens. With age, NETosis decreases and leads to delayed pathogen clearance and increased susceptibility to infection 99.

The aging inflammatory microenvironment significantly reprograms neutrophils and in turn affects multiple organs throughout the body. Aging neutrophils cannot efficiently clear inflammation but instead induce aggravated telomere damage and cellular senescence in organ parenchymal cells through the release of matrix metalloproteinases (MMPs) and ROS, resulting in chronic tissue and organ damage 100. Enhanced reverse trans-epithelial migration of neutrophils can be observed in aged mice. Due to the accumulation of mast cells expressing C-X-C chemokine ligand-1 (CXCL-1), neutrophil C-X-C chemokine receptor-2 (CXCR-2) was desensitized and lost neutrophil-directed motility at epithelial junctions, resulting in neutrophil re-entry into the circulation and causing distant lung tissue damage 101.

In summary, aging-related neutrophil hypofunction delays pathogen clearance and injury repair, increases local inflammation, and exacerbates aging-related inflammatory diseases 102. Further exploration of the interactions between neutrophils and multiple inflammatory microenvironments may help explore the underlying mechanism of aging-related multiorgan dysfunction syndrome.

### Other myeloid cells

In contrast to macrophages and neutrophils, the functional alterations and mechanisms of aging in other myeloid cells still lack in-depth studies. For example, aging DCs are significantly less efficient in cross-presenting antigens and activating CD8^+^ T cells 103, leading to a decrease in adaptive immune responses in aged hosts 104. However, it is still controversial whether different DC subtypes exhibit altered antigen-presenting ability 105. Studies in human patients have found that eosinophil degranulation capacity is significantly reduced in elderly patients, which may explain the differences in disease manifestations and drug sensitivity of airway inflammation in older and younger patients 106, but its role in diverse aging-related inflammatory diseases remains poorly understood. Activation of NF-κB, a key transcription factor in the cellular response to injury and stress, partially causes a significant increase in the proportion of MDSCs in the bone marrow and spleen of aged animals, thereby suppressing the immune response and leading to an impaired response to stress 107. Currently, the role of different subpopulations of myeloid cells in aging-related diseases remains unclear due to low cell abundance, and future omics tools such as single-cell sequencing may better explore the potential roles and mechanisms of myeloid cell subpopulations in aging.

### Interaction among myeloid cells and nonimmune systems

As one of the main features of cellular senescence, the production and secretion of SASPs not only triggers local inflammation but also induces infiltration of immune cells such as macrophages, neutrophils, NK cells and T cells 100, 108, 109. The surveillance and clearance of senescent cells by immune cells is crucial for preserving tissue homeostasis and preventing hazardous inflammation 108, 110. Senescent cells accumulate as a result of age-related immune system degradation, which may cause age-related functional decline and diseases 111. Elimination of senescent cells by gene editing prevents or delays tissue dysfunction and extends healthy lifespan, suggesting that immunosurveillance and clearance of senescent cells can help ameliorate aging-related diseases 112, 113.

The hypothesis that an aging immune system can drive solid organ aging is directly evidenced by the study from Yousefzadeh et al 47. The investigators established a mouse model of premature immune failure from deletion of *Ercc1* in hematopoietic stem cells and observed that along with aging of the immune system, nonlymphoid organs also showed increased aging and injury, whereas transplantation of immune cells from young mice blocked organ aging 47. In liver studies, infiltration of macrophages and NK cells in aged liver can inhibit hepatocyte DNA synthesis and liver regeneration by producing excess interferon-γ (IFN-γ), while depletion of such cells can significantly improve liver regeneration 114. In a mouse model of acute liver injury (ALI), the telomeres of hepatocytes are highly susceptible to oxidative damage caused by neutrophil infiltration, and depletion of neutrophils reduces telomere dysfunction and cellular senescence in hepatocytes 100. Therefore, the aging immune system can drive systemic aging and may serve as a therapeutic target to extend healthy lifespan.

Senescent nonimmune cells can also regulate the functional state of immune cells. scRNA-seq analysis revealed that the age-related changes in the number and transcriptional characteristics of alveolar macrophages are regulated mainly by the alveolar microenvironment but not by circulating growth factors and cells 76. During aging, senescent cells gradually accumulated in the liver and visceral white adipose tissue may induce macrophage proliferation with SASPs secretion 115. The interaction between immune and nonimmune cells in the aging process is still not sufficiently studied. A better understanding of the interaction during aging will help to develop new therapeutic interventions to eliminate harmful senescent cells and maintain the youthfulness of the organism.

## Myeloid cells and aging-related liver diseases

**Figure 3:**
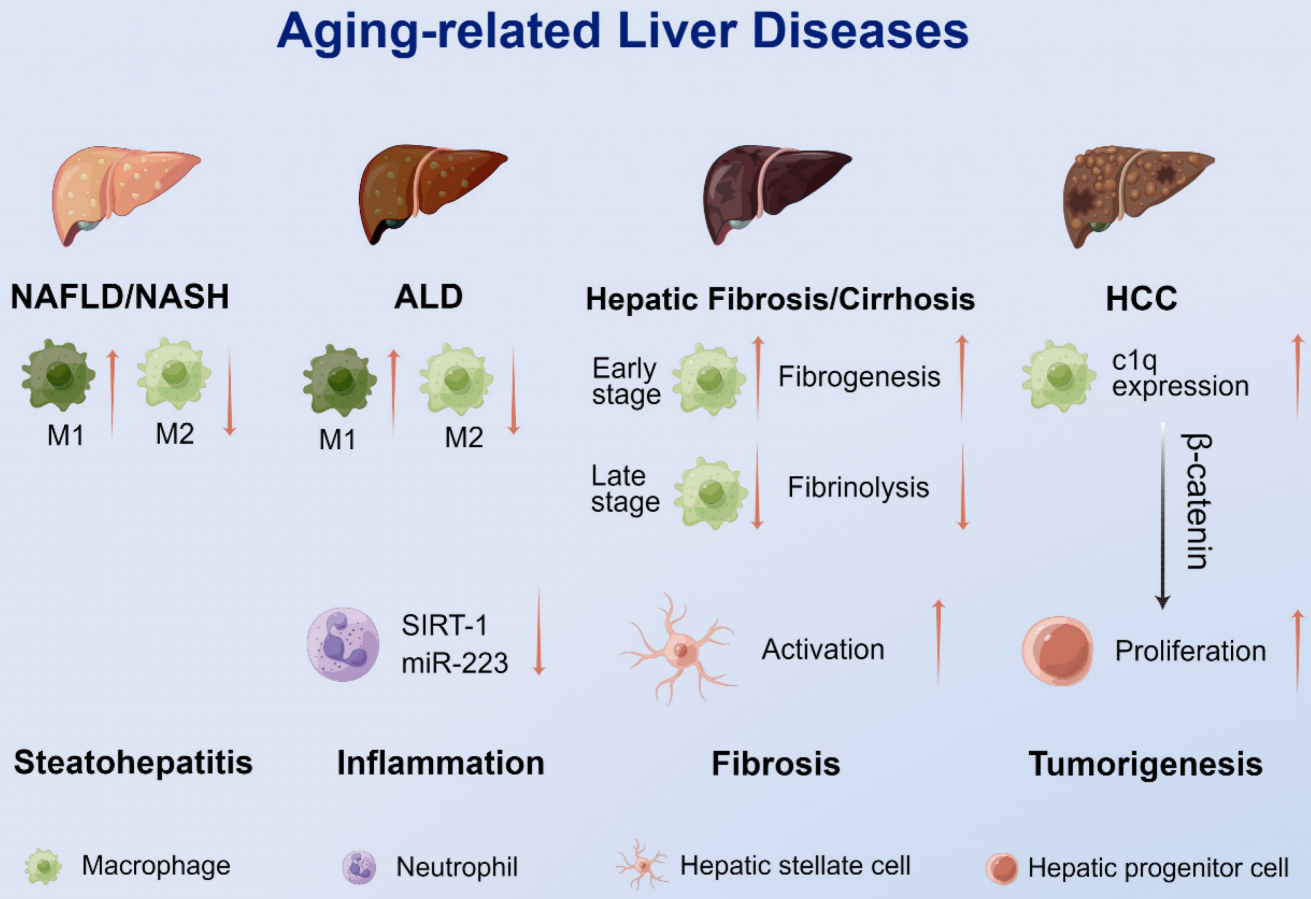
The roles of myeloid cells in aging-related liver disease. An increase in M1 macrophages and a decrease in M2 macrophages were observed in NAFLD and ALD. Reduced SIRT-1 expression in aged neutrophils suppressed miR-223 expression, causing more severe inflammatory injury in ALD. Increased macrophage infiltration and fibrogenesis can be observed in the early stage of chronic injury, while decreased macrophage infiltration leads to decreased fibrinolysis and promotes liver fibrosis through activation of HSCs in the late stage. During aging, macrophages express increased complement C1q which promotes the proliferation and dedifferentiation of HPCs and tumorigenesis through the β-catenin pathway. ALD: alcoholic liver disease; HCC: hepatocellular carcinoma; M1: M1 macrophages; M2: M2 macrophages; miR-223: microRNA-223; NAFLD: nonalcoholic fatty liver disease; NASH: nonalcoholic steatohepatitis; SIRT-1: sirtuin-1.

The immune system plays an integral role in developing liver diseases 116. There is growing evidence supporting that aging immune cells, especially myeloid cells, directly or indirectly contribute to liver aging and increased susceptibility to aging-related diseases, including NAFLD/nonalcoholic steatohepatitis (NASH), ALD, liver fibrosis/cirrhosis, hepatocellular carcinoma (HCC) and comorbidities involving the liver (Figure 3).

### Nonalcoholic fatty liver disease

NAFLD is the leading cause of chronic liver disease worldwide 117. Compared to young people, elderly people are more likely to progress to NASH 118. The mechanisms underlying the development of aging-related steatosis are not fully understood and may be attributed to the accumulation of toxic free fatty acids caused by mitochondrial dysfunction 119, reduced autophagy 35, 120 and endoplasmic reticulum stress 121 in aged hepatocytes.

During the disease process of NAFLD, macrophages accumulate in the liver through C-C motif ligand-2 (CCL-2)/CCR-2 chemotaxis 122. M1 macrophages promote the development of aging-related NAFLD/NASH, while M2 KCs promote the apoptosis of M1 KCs and reduce hepatocyte apoptosis and steatosis, thereby alleviating the disease progression of NAFLD 123. Fontana et al. 124 used an HFD to trigger NAFLD in mice of different ages. Liver injury occurred mostly in older mice with more pronounced M1 macrophages in the liver and adipose tissue than in younger mice. Similarly, a significant positive correlation between CD163, a marker of M1 macrophages, and the severity of NAFLD was observed in human patients 125. Therefore, the infiltration and polarization of macrophages influences the course of aging-related NAFLD/NASH, and therapies targeting macrophages may improve the incidence and severity of this disease in the elderly population.

### Alcoholic liver disease

ALD is one of the leading causes of death from liver diseases, and its pathological changes include a series of processes, including steatosis, steatohepatitis, cirrhosis, and HCC 126, 127. In recent years, the incidence of ALD has tended to be stable, but the incidence of alcohol-related cirrhosis and HCC and the need for liver transplantation are continuing to rise 128. Elderly people are prone to excessive alcohol consumption, and the risk of alcoholism is further increased due to altered metabolism or tobacco and illicit drug use 129. Old individuals tend to have a chronic low-grade inflammatory state, as evidenced by elevated circulating levels of pro-inflammatory cytokines and local infiltration of inflammatory cells, which further exacerbates ALD 130, 131.

KCs are the major defense of hepatic innate immunity. Increased intestinal permeability and high levels of endotoxin in portal blood were found to lead to the activation of KCs 132. KCs play key roles in early alcohol-induced liver injury by recognizing endotoxins in the portal circulation and inducing an innate immune response through polarization to the pro-inflammatory M1 phenotype 132. Wan et al. 133 found that promoting the polarization of KCs to the anti-inflammatory M2 type prevented alcohol-induced hepatocyte steatosis and apoptosis. A high M2/M1 ratio may be a protective feature of ALD 123.

Emerging evidence supports liver infiltration of neutrophils as a key mechanism in promoting ALD, possibly by producing ROS and inflammatory mediators 134, 135. SIRT-1 is a recognized antiaging protein, and knockdown of SIRT-1 may exacerbate ALD 136. Our recent study found that neutrophilic SIRT-1 expression was significantly downregulated in aged and ethanol-fed mice, and myeloid cell-specific* sirt-1* knockout mice had more severe ALD 89. In addition, downregulation of SIRT-1 decreased the expression of anti-inflammatory/antifibrotic microRNA-223 in neutrophils 137, 138, leading to an increase in the production of ROS and inflammatory mediators, which ultimately resulted in acute alcoholic liver injury in alcohol-fed mice and patients with chronic alcohol consumption.

Recent study from Ma and Gao et al. 139 have expanded existing knowledge on the roles of hepatic neutrophils in mediating liver injury. Based on the extent of inflammatory cell infiltration, severe alcoholic hepatitis was divided into high intrahepatic neutrophils with low CD8^+^ T cells (Neu^hi^CD8^lo^) and Neu^lo^CD8^hi^ subtypes, and the effects of the neutrophil cytosolic factor-1 (NCF-1)/SIRT-1/AMPK axis on lipid metabolism and the NCF-1/p-38 MAPK/miR-223 pathway on alcohol-induced inflammation and fibrosis were further revealed 139. Consistent with the notion that neutrophils in young adults have a greater capacity to release ROS 95, Neu^hi^CD8^lo^ patients were relatively younger and had more severe liver injury compared with those with Neu^lo^CD8^hi^. However, considering the inclusion of patients who are mostly middle-aged in the two groups, it remains unknown whether hepatic neutrophils exert pro-inflammatory effects through similar pathways in the truly aged population. But one thing is clear that treatments targeting neutrophil must be a promising direction for future translational research, which is expected to improve the prevention and treatment of ALD as well as its complications.

### Liver fibrosis and cirrhosis

Liver fibrosis, which can progress to cirrhosis, HCC, and eventually liver failure, is one of the main outcomes of various chronic liver diseases. Liver fibrosis is a dynamic and reversible process of wound repair, including progression and regression stages 140. Various cytokines and chemokines secreted by macrophages, including TGF-β, TNF-α, IL-1β and CCL-2, activate HSCs and enhance myofibroblast proliferation through NF-κB-dependent signaling pathways, eventually resulting in liver fibrosis 141.

The incidence of liver fibrosis increases with age and is typically characterized by age-related changes in macrophage infiltration. In the early stages of chronic injury, macrophages infiltrate more in the aged liver than in the young liver, which promotes inflammation and fiber formation. In the later stages, macrophage infiltration of the aged liver is reduced and insufficient to adequately lyse fibers, thereby exacerbating liver fibrosis 142. The direction of macrophage polarization may also influence the liver fibrosis process. Mohammed et al. 143 found that markers of M1 macrophages, pro-inflammatory cytokines (TNF-α, IL-6 and IL-1β), and markers of fibrosis were significantly upregulated in aged mice with necroptosis-induced liver injury. To inhibit macrophage recruitment, a recent clinical trial using CCR-2/CCR-5 antagonists found that, as compared to placebo, twice as many participants achieved fibrosis improvement and no exacerbation of steatohepatitis 144. Subgroup analysis showed that the therapy was equally effective in patients under and over 56 years of age, indicating its potential therapeutic value in aging-related liver fibrosis 144.

### Hepatocellular carcinoma

HCC occurs mostly on the basis of viral infection or noninfectious chronic hepatitis 145. These chronic inflammatory conditions create a pro-tumorigenic microenvironment that is an important factor in inducing the transformation of hepatocytes into cancer cells 146. Advanced age is a high-risk factor for HCC due to the constant low-grade inflammation that comes with aging 44. Research from Ho et al. 147 revealed that aged mice with knockout of hepatocyte β-catenin had an inflammatory microenvironment contribute to the development of HCC. C1q, a complement released from macrophages in the inflammatory microenvironment, is thought to activate the nonclassical pathway of β-catenin in periportal hepatic progenitor cells (HPCs), promoting the proliferation and dedifferentiation of HPCs and thus inducing hepatocarcinogenesis. C1q inhibitors blocked the β-catenin pathway in HPCs and HCC but not the classical pathway in normal hepatocytes. The above mechanism was also verified in liver specimens from patients with chronic hepatitis. Therefore, C1q in macrophages may be a new target for blocking carcinogenesis in elderly patients.

### Other liver diseases

In addition to the abovementioned diseases, aging may also affect liver diseases pertaining to the dysregulation of proliferation, healing, and tolerance. During severe acute injury, the liver regenerative capacity may be impaired, leading to ALI and even acute liver failure (ALF). Compared with young people, ALI in elderly individuals is more likely to develop into ALF with a worse prognosis. Our recent study, using the thioacetamide (TAA)-induced ALI mouse model (TAA-ALI), demonstrated that aging can polarize macrophages to the M1 phenotype through the secretion of inflammatory factors and SASPs and thus exacerbate liver injury 35. Macrophages are also involved in postoperative ischemia and reperfusion (IR) liver injury. In aged IR mice, NLRP3 activation can be observed in the liver and macrophages, while knockdown of the STING-NLRP3 axis in macrophages eliminates the deleterious role of aging in exacerbating intrahepatic inflammation and IR injury 148. Other aging-related liver comorbidities, such as multiorgan dysfunction, may also be regulated by KCs 149, and more studies are needed to reveal the impact of the immune system on aging-related liver diseases.

## Promising therapeutics targeting aging

As the population ages, the rise of aging-related liver diseases is not just a medical issue but also a significant social issue without new preventive and curative therapeutics specifically for not only young and middle-aged populations but also elderly people. In this scenario, several strategies focused on the aging immune system, a novel therapeutic target, to treat aging-related liver diseases have shown promising prospects, and some of them may have potential in clinical application (Table 1).

### Senolytic therapy

As mentioned above, aging-related immune senescence, which leads to a reduction in the phagocytosis capacity of macrophages and neutrophils, may result in the accumulation of senescent cells and increased susceptibility to diseases. Senolytic therapy was designed to compensate for immune clearance and maintain organ rejuvenation. Using a hypothesis-driven strategy that targeted senescent cell antiapoptotic pathways which were more highly expressed by senescent than nonsenescent cells, the first generation senolytic medicines dasatinib, quercetin, fisetin, and navitoclax were discovered 150. In preclinical models, senolytics prevent or alleviate cardiovascular 151, liver 119, lung 152, kidney disorders 153 as well as complications of organ transplantation 154, age-dependent fracture healing 155 and disc degeneration 156.

Preclinical studies of senolytic therapies for liver diseases have shown promising results. The classic senolytic cocktail of dasatinib plus quercetin (D + Q) reduced overall hepatic steatosis, thus alleviating aging-related NAFLD 119 and CCl_4_-induced liver fibrosis 157 in mice. Fisetin has also been shown to improve APAP-induced hepatotoxicity by promoting autophagy and inhibiting inflammasome activation 158. In addition to chemical drugs, chimeric antigen receptor (CAR) T cells that target senescent cells have also shown promising results in ameliorating liver fibrosis induced chemically or by diet 159. However, senolytic therapy is not a panacea. Research from Cheng et al. 160 showed that ABT263 (navitoclax) may impair liver regeneration following partial hepatectomy through elimination of senescent HSCs, which induces multiple signaling pathways to stimulate liver regeneration by secretion of IL-6 and CXCR-2 ligands. Moreover, D + Q was ineffective against age-associated NAFLD-induced HCC 161. Therefore, until the pharmacological mechanism, efficacy, and safety of senolytics are well clarified, there is still a long way to go before senolytic therapies can be clinically applied in aging-related liver disease.

### Caloric restriction

CR is typically a daily decreased nutrient uptake intervention without malnutrition 162. CR extends the lifespan of various model organisms ranging from yeast, worms and flies to mice and primates 163 by regulating hallmarks of aging, including deregulated nutrient sensing, cellular senescence, stem cell exhaustion and altered intercellular communication 162. Recently, scRNA-seq analysis revealed that aging may be delayed by CR by reversing the aging-disturbed immune ecosystem, which has excessive pro-inflammatory ligand-receptor interplay 64 and extends health lifespan 164. In humans, a two-year study of CR identified a reduction in the rate of living and systemic oxidative stress and improved biomarkers of aging 165. CR also leads to a marked improvement in glucose metabolism 166 and reverse fatty liver caused by saturated fat overeating 167.

Given that a healthy lifestyle requires sustained efforts and discipline, it is easier said than done for routine application of CR. Therefore, several pharmaceutical compounds or dietary supplements that mimic the effects of CR are being explored. Metformin, previously used as an antidiabetic drug, is viewed as a CR mimetic via activation of AMPK 168. Studies have shown evidence of metformin attenuating aging hallmarks 169 and ameliorating age-related changes in LSECs via AMPK and endothelial nitric oxide pathways, which may improve liver insulin sensitivity, especially in old age 170. In addition to metformin, resveratrol, another classic CR mimetic, has shown promising results in preclinical studies pertaining to ALI 171 and NAFLD 172. However, current evidence, including results from clinical trials, does not support supplementation with resveratrol for the management of NAFLD 173-175. We believe that healthy lifestyle and dietary habits may contribute more to delaying aging and improving susceptibility to aging-related diseases than the use of synthetic drugs 176. Individualized strategies that combine genetic alteration, disease pathology and lifestyles will bring the greatest benefit to patients.

### Microbiological therapy

Gut microbes occupy the interface between the external environment and the host 177, which significantly influences human aging and diseases 178. Dysbiosis is characterized by an imbalance in the microbiota including local distribution, functional composition and metabolic activities 179. Aging-related dysbiosis of the gut microbiome was proven to contribute to a global inflammatory state and diseases in the elderly 180 through chronic upregulation of pro-inflammatory mediators (TNF-α and IL-6), thus serving as a catalyst for fueling inflammaging 44. These mediators activate many signaling pathways influencing immune function, leading to a gradual deterioration of the immune system, known as immunosenescence 181. Accumulating evidence suggests that both inflammaging and immunosenescence are responsible for most aging-related diseases, including but not limited to disorders pertaining to the cardiovascular 182, neurological 183, respiratory 184 and digestive systems 143.

Changes in the composition and function of the gut microbiota have profound effects on the development and management of liver disorders, including steatosis 185, cirrhosis 186, liver failure 187 and even HCC 188. Recently, new promising probiotics, such as Saccharomyces boulardii, have been discovered and have potential therapeutic effects in ALI, ALF and liver fibrosis 189. Living materials of a hierarchy-assembled dual probiotic system containing bulgaricus and Lactobacillus rhamnosus GG, fabricated by Chen et al. 190, effectively prevented cholestatic drug-induced liver injury through inhibition of hepatic bile acid synthesis and facilitation of bile acid excretion. Fecal microbiota transplantation (FMT), which involves the transfer of donor stool into recipients, is increasingly being explored as a potential treatment in gastric intestinal and liver diseases 191. Clinical trials supported the safety of FMT and have shown improvement of survival and clinical severity in patients with severe alcoholic hepatitis 192, 193. However, FMT can be lethal for elderly people because of serious complications, such as infections from multidrug-resistant organisms 194. Therefore, FMT should be used with appropriate monitoring and careful donor selection in treating aging-related liver diseases in elderly population. With a further understanding of the crosstalk among the microbiome, aging and disease, individualized treatment focusing on different disease conditions and populations will better balance the efficacy and safety when being administered in aging-related liver diseases.

## Conclusion and outlooks

Aging is the process of gradual decline of organisms over time. Liver, the largest digestive and immune organ in the body, also deteriorates with age, increasing the risk of chronic liver diseases. Immune surveillance and clearance maintain the homeostasis and normal functioning of the liver, whereas the aging immune microenvironment inappropriately releases excessive inflammatory factors, causing and exacerbating various aging-related liver diseases.

There are still many unknowns in the field of liver aging, such as whether immune cells that form different liver structures affect the course of aging-related diseases differently, the interaction between immune cells and nonimmune mesenchymal cells and the functional or metabolic changes they cause, and the unique roles of low-abundance myeloid cells such as DCs and eosinophils in liver aging. In addition, the lack of clinical exploration has limited the translation of results from animal research. Distinguishing physiological aging from age-related conditions can be challenging, and more studies are needed to assess the effects of aging on the liver prior to the onset of age-related conditions to separate the mechanisms of aging from the manifestations of the pathological conditions themselves. In the future, as new technologies become more available, multiomics analysis at the single-cell level will further reveal the roles immune cells play in regulating aging at the organ and even systemic level, providing an updated understanding and individualized treatment strategies for aging-related diseases.

## Figures and Tables

**Table 1:** Clinical trials with anti-aging strategies for liver diseases.

Strategy	Study ID	Indication	Regimen	Status
Senolytic Therapy	NCT00382668	Liver diseases	Dasatinib	Completed
	NCT00459108	HCC	Dasatinib	Terminated
	NCT02143401	HCC	Navitoclax	Active, not recruiting
Caloric Restriction	NCT04230655	NAFLD	Low energy diet	Recruiting
	NCT05041673	Fatty liver	Metformin	Active, not recruiting
	NCT04972396	NASH	Metformin	Recruiting
	NCT04033107	HCC	Metformin	Recruiting
Microbiological Therapy	NCT03796598	Liver cirrhosis	FMT	Recruiting
	NCT04594954	NAFLD	FMT	Recruiting
	NCT04758806	Alcoholic hepatitis	FMT	Recruiting
	NCT05007470	ALD	VSL #3 Capsule	Recruiting

**Aberrations:** ALD: alcoholic liver disease; FMT: fecal microbiota transplantation; HCC: hepatocellular carcinoma; NAFLD: Nonalcoholic fatty liver disease: NASH: nonalcoholic steatohepatitis.

## Abbreviations

ALD: alcoholic liver disease
ALI: acute liver injury
ALF: acute liver failure
AMPK: Adenosine 5'-monophosphate activated protein kinase
ATG5: autophagy-associated protein 5
BMDMs: bone marrow-derived macrophages
CLP: common lymphoid progenitor
CMPs: common myeloid progenitor cells
CR: caloric restriction
DCs: dendritic cells
DSBs: DNA double-strand breaks
EMPs: erythro-myeloid progenitors
FMT: fecal microbiota transplantation
GMPs: granulocyte macrophage progenitor cells
HCC: hepatocellular carcinoma
HDAC: histone deacetylase
HFD: high-fat diet
HGF: hepatocyte growth factor
HPCs: hepatic progenitor cells
HSCs: hepatic stellate cells
ICAM-2: intercellular adhesion molecule-2
IL-6: interleukin-6
IR: ischemia and reperfusion
KCs: kupffer cells
LPS: lipopolysaccharide
LSECs: liver sinusoidal endothelial cells
MANF: mesencephalic-astrocyte-derived neurotrophic factor
MAPK: mitogen-activated protein kinase
MDSCs: myeloid-derived suppressor cells
MMPs: matrix metalloproteinases
mtDNA: mitochondrial DNA
NAFLD: nonalcoholic fatty liver disease
NASH: nonalcoholic steatohepatitis
NCF-1: neutrophil cytosolic factor-1
NETs: neutrophil extracellular traps
NK: natural killer
PGE2: prostaglandin E2
ROS: reactive oxygen species
SASP: senescence-associated secretory phenotype
SIRT-1: sirtuin-1
TIM-4: T-cell immunoglobulin mucin receptor-4
TNF: tumor necrosis factor
